# A complex challenge with unclear improvement: the need for involvement, contextualization and facilitation when managers implement a leadership model

**DOI:** 10.1108/LHS-05-2022-0055

**Published:** 2022-10-05

**Authors:** Maria Lindberg, Bernice Skytt, Magnus Lindberg, Katarina Wijk, Annika Strömberg

**Affiliations:** Department of Caring Sciences, University of Gävle, Gävle, Sweden; Centre for Research and Development, Uppsala University, Uppsala, Sweden and Department of Public Health and Caring Sciences, Uppsala University, Uppsala, Sweden; Department of Caring Sciences, University of Gävle, Gävle, Sweden and Department of Public Health and Caring Sciences, Uppsala University, Uppsala, Sweden; Department of Caring Sciences, University of Gävle, Gävle, Sweden; Centre for Research and Development, Uppsala University, Uppsala, Sweden; Department of Public Health and Caring Sciences, Uppsala University, Uppsala, Sweden and Department of Occupational Health Sciences and Psychology, University of Gävle, Gävle, Sweden; Faculty of Health and Occupational Studies, University of Gävle, Gävle, Sweden and Department of Business and Economics studies, University of Gävle, Gävle, Sweden

**Keywords:** Change management, Health services, Implementation, Leadership, Organisation and administration, Qualitative research

## Abstract

**Purpose:**

Management and leadership in health care are described as complex and challenging, and the span of control is known to be a key component in the manager’s job demands. The implementation of change can be a challenge in health care, and managers often have roles as implementation leaders. Little attention has been given to how managers perceive the process of implementation. Thus, this study aims to explore second-line managers’ perceptions of, prerequisites for and experiences from the implementation of changes in their manager’s work conditions.

**Design/methodology/approach:**

A grounded theory–based qualitative design was used. Data were collected from a purposive sample of nine second-line managers by individual semi-structured interviews. The three stages of initial coding, focus codes and axial coding were used in data analysis.

**Findings:**

Three thematic areas were identified: engagement, facilitation and achievement. The second-line managers’ descriptions suggest that the change work entails a complex challenge with an unclear result. Involvement, consideration for the context and facilitation are needed to be able to conduct a cohesive implementation process.

**Originality/value:**

This study findings outline that to succeed when implementing change in complex organizations, it is crucial that managers at different levels are involved in the entire process, and that there are prerequisites established for the facilitation and achievement of goals during the planning, implementation and follow-up.

## Introduction

The role of manager or leader in health-care settings is often described as complex and challenging (Figueroa *et al.*, 2019). Managers acting as implementation leaders should create prerequisites that generate a workplace predicated on the ability to influence, participate and trust; a workplace that also facilitates evidence-based practices (Castiglione, 2020). Previous research has strived to understand how to implement scientific advances into health-care settings. It is crucial that a focus is also aimed at pushing the evidence-based knowledge into routine use (Brownson *et al.*, 2017). Managers face the complex challenge of handling and leading professionals with diverse health-care giving experiences (Södersved Källestedt *et al.*, 2020). In health-care management, it has been found that structural features related to the organisational characteristics of hospitals can play a role in the measured outcomes (Dijkstra *et al.*, 2006). Another dimension that is discussed by Ovretveit is the complex role leaders have in engaging clinicians in improvements (Ovretveit, 2010). Clinical professionals may have a leadership role, but not necessarily the responsibility of a manager (Patole, 2015).

One prerequisite that has been implemented in public sector management is a span of control in which management is a functional organisation with a reasonable number of subordinates per manager (Andersson-Felé, 2008). Studies show that the span of control is a key component in a manager’s job demands (Wallin *et al.*, 2014). Furthermore, subordinates, i.e. nurses, practical nurses and nurse coordinators/specialists, have described how first-line managers (FLMs), i.e. the frontline leaders who have more than 50 subordinates, have been regarded as distant and disorganised leaders. Additionally, the FLMs themselves experienced a low degree of role clarity and substantial challenges in creating an overview (Holm-Petersen *et al.*, 2017).

Durlak and DuPre (2008) concluded that more information is needed on how various factors influence the implementation. They stress the importance of creating concrete proposals that address, e.g. a common goal for the intended change, the mapping of problems during the implementation, the development of a set of strategies for the change and the implementation plan, the integration of change in normal practice and continuous monitoring. Grol *et al.* (2013) explore the implementation of change in health care, and reveal possible reasons for adopting changes such as structural, financial or organisational obstacles, but also “the nature, the applicability of the new proposed method of working, the professionals who need to change, or the setting in which the intended change is to take place” (p. 14). However, little attention has been given to the process of implementation and how managers perceive it.

In a mid-sized region in Sweden, a leadership model was implemented to create good prerequisites for management and leadership. Good conditions included a reasonable number of subordinates for FLMs and a limited number of participants in management teams, as well as clarity in assignments and expectations (Box 1). In this region, the second-line managers were the persons who had the responsibility to lead the implementation in their own units. Having the two overarching mandates of manager and implementation leader, and managing this dual responsibility, can be challenging. Therefore, the aim was to explore second-line managers’ perceptions of, prerequisites for and experiences from the implementation of changes in their manager’s work conditions.

Box 1. The five focus areas for improvement

The implementation of improvements to rectify the managers’ conditions focused on these five areas:

1. Managers must have clarity and knowledge about vision, goals and strategy.
2. Management teams must consist of a maximum of ten persons.
3. Managers are to have a clear assignment, continuous interaction with their boss, and clarity about expected working hours and availability.
4. The scope of their authority and responsibilities must be revised.
5. Managers should generally have a maximum of 30 subordinates.

## Methods

Ethical approval was granted by the Swedish Ethical Review Authority, registration number 2019-04583.

### Study setting

The Swedish health-care system is decentralised and divided into 21 regions that are led by elected council members. Each region has the responsibility to provide its residents with good-quality health and medical care, but also preside over collective functions such as public transport, regional development and culture. There are about 285,000 residents in the region involved in this study, and it has about 6,000 employees in the health-care sector. A second-line manager is responsible for both patient safety and the work environment of the personnel. As an employer, a region must according to Swedish law “systematically plan, direct and monitor activities in a manner that ensures that the work environment meets the prescribed requirements for a good work environment”. A review was carried out, and challenges the managers encountered in their work conditions were identified. A need was identified to strengthen the conditions for managers so they could practice and achieve the desired management and leadership mandates. A leadership model that focused on the region’s and the operation’s goals as well as one that expressed important driving forces and competencies that should characterise the leadership in the region was developed. Thereafter, external facilitation was arranged before implementation. The implementation of improvements to rectify the managers’ conditions focused on the five areas presented in Box 1.

### Sampling and study participants

A quota sampling strategy (Robinson, 2014) was used to identify participants among second-line managers. With the information about their managers’ recent spans of control, we divided them into three groups. The first group was characterised as previously having an explicit need to change the managers’ spans of control so they would be able to comply with the requirements. The second group included previous borderline cases needing change, and the third included managers who were already complying with the requirements. By recruiting from each stratum, we could ensure that diverse characteristics related to the study phenomenon were represented in the sample. Email invitations for interviews and study information were sent to 14 potential participants from a total of 20 second-line managers (by MaL). A follow-up telephone call was made to provide additional information verbally. Informed consent and interviews were obtained from nine second-line managers. The sample included five males and four females who were 49–64 years old (mean 55.7) and had between 8 and 33 (mean 19.3) years of experience in managerial positions. They had on average been working 3.1 years in their current position (range 1–5.5). Stated reason for not participating was lack of time.

### Data collection

Data were collected using a qualitative approach over a period of nine months until September 2020. In-depth, semi-structured interviews using open-ended questions were conducted (by BS, a female experienced qualitative researcher) face to face with individual participants at their workplace. Open-ended questions were used to capture statements of relevance for the aim of study. Examples of probing questions are “Please, describe how you have perceived your role in the implementation” and “Please, describe how you have worked with the actual implementation”. Follow-up questions were used to get a deeper understanding of given statements. The length of the interviews varied from 42 to 88 min (mean 67.6 min and median 68 min) and all interviews were audio-recorded. The recordings were then transcribed verbatim.

### Data analysis

**Figure 1.**
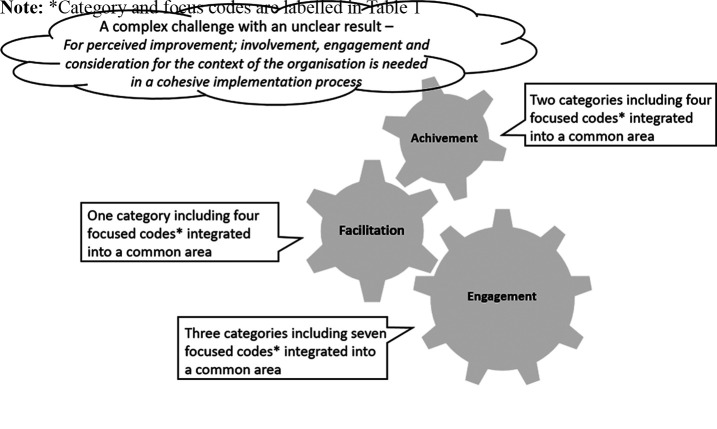
Visualization of the core category and thematic areas in relation to each of the six categories and the 15 focused codes

Consistent with grounded theory methods (Charmaz, 2006), data collection and analyses were concurrent. A constructivist approach was used, and the terminology applied was based on a description by Hoare *et al.* (2012). In the first stage of the analysis, the initial coding was performed line-by-line and led to the open coding and construction of a series of codes. These codes used participant language that crystallized and condensed meanings, which transformed into categories. In the second stage, the initial codes were gone through, sorted and synthesized to identify focused codes that were considered to have theoretical reach. The third stage ensured integration and comprised axial coding that would relate each category with the others. The axial coding procedure provided a way to identify relationships between categories in thematic areas. Constant comparison of data to data and then data to categories was used until saturation was reached and preliminary results was discussed with participants from the organization. A storyline technique was then used to discover the core category of the theoretical construct exploring perceptions of, prerequisites for and experiences from the implementation of changes in manager’s work conditions. The core category and the thematic areas are visualized in Figure 1. The analysis was conducted (by Maria Lindberg and Annika Strömberg) using Microsoft Word documents. All researchers met several times through this iterative process.

## Results

The participants’ perceptions of, prerequisites for and experiences from implementing changes in relation to the five focus areas revolves around three thematic areas: engagement, facilitation and achievement. They described the work to accomplish change, as being a complex challenge with an unclear effect. Additionally, to be able to conduct a cohesive implementation process, they expressed the need to be involved and engaged, to have consideration for each unit’s context and to receive facilitation assistance (Table 1). The core category and its construct in relation to each of the categories and the focused codes are displayed in Figure 1. Below, the three thematic areas are presented and describes the categories within each thematic area in line with the method used.

### Engagement in an important change and an established model in a complex organisation

“I completely understand that a person needs certain framework for it to (.) work. But to go in with (.) a (.) square decision (.) in an organisation that is made of triangles and circles and squares is rather (.) poorly thought out from a management perspective. Because it is not possible to put (.) square pegs in round holes.” (IP6)

The second-line managers stated that top management made it clear how investments are imperative to improve the conditions for FLMs. The participants agreed that a focus on the FLMs prerequisites is of great importance because prerequisites play a significant role and serve an essential purpose in the organisation, e.g. they lay the foundation so the FLMs can give support to the staff. They also stated that good prerequisites are important for the FLMs themselves.

The participants perceived that they had not been able to influence the content of the five focus areas for change, even though they had participated in conversations during the preparation work that was done to identify and determine possible solutions. They also perceived that no feedback had been given during the process. Thus, they perceived the process as being top-down management, which left them uncertain about the motive of the content. Additionally, they described a lack of information regarding the validity of the scientific and empirical content. To be able to better facilitate the implementation, the participants explained how they should have been involved before the discussions on the needed changes, e.g. content and maximum numbers.

When the decision regarding the five focus areas was presented to the second-line managers, the common perception among the participants was that the focus was on only two of them: a maximum number of subordinates and a maximum size of the management teams. The participants reported that top management had declared that the introduction of assistant FLMs with a responsibility for personnel was the solution for the number of subordinates. The participants expressed that they did not think that a maximum number of subordinates would solve the situation for the FLMs. They regarded the FLMs’ conditions as being more complex because the handling of cultures, systems and individuals differs between units. They expressed the desire and the need to have been included in forum discussions regarding the content of the five focus areas together with other second-line managers, so that a more accurate handling of their FLM’s specific conditions could have been achieved.

### The individual was left on their own to facilitate the process

“I thought it was arduous that I had to think about everything by myself […] it would have made my communication with the management team or with the personnel easier if I could say that this is an assistant manager who should do these things.” (IP1)

Although the participants expressed their opinion that the decision regarding the five focus areas was rather unproblematised, they began the processes for change in their units. In these processes, the participants described a need for different types of support that were not always fulfilled, e.g. lack of support from designated facilitators and the need for dialogue on how to implement the changes. On the contrary, the participants described how they did receive support when needed from, for example, human resources. Without having the possibility to exchange thoughts and concerns with others, e.g. in discussion forums to discuss the change work, they expressed that they were not given the best prerequisites to be of good support to the FLMs. The internal support within their own management team was considered crucial for the participants when leading the change work, i.e. to have continuous dialogue, to find ways to adapt solutions in accordance with the FLMs’ needs and to analyse possible consequences of change in their own unit. The participants also told that because of the lack of a job description for assistant FLMs, they had to design one. The only information they had received was that the assistant FLM should have responsibilities for personnel, but not to what extent. Because of the lack of support from appointed external facilitators, the participants found it difficult to defend and motivate the intentions of the focus areas concerning the maximum number of 30 subordinates for FLMs and that their own management teams must consist of a maximum of 10 persons.

### Different methods of implementation and uncertainty regarding the achievement of improved conditions for managers

“Yes. I expect that we will follow up on what we have done […] we haven’t discussed the question of content, it is more a quantitative follow-up […] there is no change that will be 100% correct right away, without our having to find a way to evaluate and fix what needs fixing.” (IP4)

There were differences among the participants regarding the mandatory or recommended nature of the five focus areas. Some were under the impression that they were free to implement the changes that they themselves considered necessary. Those who considered the decisions mandatory, described how because those aspects were not included in the five focus areas for change, they could not implement the changes they actually needed, such as the need to work with aspects that develop the unit and create better prerequisites for the FLMs to lead their unit for change. The participants explained that they had the ambition to implement the changes according to the decision, but they had different approaches on how to accomplish them at their different units. The most common change was to replace the function of the clinical nurse leader with an assistant FLM who was also assigned a shared responsibility for the personnel together with the FLM. In those cases, the personnel were usually divided between the two managers based on occupational categories. Several of the participants disregarded the limit of ten representatives in the management team, as they would have preferred to focus on roles or assignments instead of numbers.

The participants feared there would be difficulties associated with a shared leadership. They were uncertain over how the subordinates would perceive the roles and responsibilities with the assistant FLM as their manager. Additionally, they expressed how shared leadership increases the need for coordination and can make it difficult to grasp the whole picture. However, the participants who had been prepared to meet the challenge with a maximum number of subordinates, experienced the implemented change as providing relief for the FLM. Working together as two was considered to create stability, enable dialogue and facilitate the possibility to always have one manager present at the unit during business hours. Even though the number of subordinates was decreased for the FLMs, the participants were uncertain whether the FLMs’ conditions had actually been improved. They described a lack of feedback and learning from the implemented changes. The focus of the follow-up was perceived to have been on how many units had reached the designated maximum number of subordinates. They, therefore, described a need to evaluate whether the implemented changes had led to improved conditions for the FLMs when discharging their responsibilities.

## Discussion

The results indicate that the participants had a common perception regarding the need to change the conditions for the FLMs so they could better discharge their responsibilities. Overall, the participants’ perceptions were that the change work entailed a complex challenge with an unclear effect. They expressed a need to be involved and engaged, to have consideration for each unit’s context and receive assistance from an appointed external facilitator to be able to conduct a cohesive implementation process and perceive improvement. We interpret this to suggest that the engagement was straightforward initially; especially with structural factors such as the number of managers. However, as previously described by Durlak and DuPre (2008) and Grol *et al.* (2013) engagement in the intended change and mapping problems were not straightforward to the participants during the implementation. When implementing change in complex organisations, adaptation to the specific context is important to engage the individuals who will be affected by the change (Damschroder *et al.*, 2009). The results are also in line with the Promoting Action on Research Implementation in Health Services (PARIHS) framework for implementation proposed by Stetler *et al.* (2011) that found facilitation, context and environment to be the crucial factors for successful implementation.

Although there was a general consensus over the need to change the conditions for FLMs, the content of the five focus areas was controversial. Context-dependent aspects were not taken into consideration. In terms of implementation, such disagreement could be considered as the organisation’s readiness for change (Weiner, 2009), and in particular to the second-line managers’ psychological and behavioural preparedness to make the changes (Weiner *et al.*, 2009). Patri *et al.* (2021) suggest effective leadership as an essential ingredient when it comes to implementation in health care. They describe it as important to look for leadership dispositions like modesty, accountability, transparency, proficiency and scientific thinking in individuals to ensure readiness for driving change management programmes. The participants spoke much about the prerequisites for FLMs from a leadership perspective. This indicates that they might have expected the change work to include prerequisites regarding both management and leadership before they were presented with the five focus areas. According to previous research, to reach a common goal with the intended change work, communication is of great importance (Durlak and DuPre, 2008; Grol *et al.*, 2013; Fixsen *et al.*, 2009). The focus in the change work had been on the two structural prerequisites regarding maximum numbers and not on the three areas that can be considered to be connected with the managers’ knowledge and duties. This implies that consensus was lacking between the top management and second-line managers regarding their views over the needs and goals that they wanted to reach with the implementation in their setting at that time. To succeed with implementation, such consensus is of importance (Weiner *et al.*, 2009). Further, Jacobs *et al.* (2014) conclude that engagement of all participants involved in a change process into an agreed approach for the change are a useful addition to the tools available to project managers. If those involved have an opportunity to influence the change, they are prepared for it and understand the value of the intended change; the likelihood of succeeding with the change is increased (Nielsen and Berthelsen, 2019). Furthermore, Grol *et al.* (2013) found that a combination of top-down and bottom-up approaches are often needed to achieve sustainable change. However, the focus on the structural prerequisites regarding maximum numbers, and especially the one regarding the FLM’s span of control, is highly relevant as it is a vital factor in the demands experienced by health-care managers (Wallin *et al.*, 2014; Holm-Petersen *et al.*, 2017).

When the participants were expected to implement changes, they expressed a feeling of being left on their own. They did, however, take on the task of being the implementation leader within their units. As an implementation leader, a person has five specific roles, i.e. planner, coordinator, facilitator, motivator and evaluator (Urquhart *et al.*, 2018). Sometimes, the participants considered it difficult to defend and implement the changes. This became particularly challenging because they were not provided with the support that had been arranged from external facilitators. In the PARIHS framework, the facilitation is described as a core construct to enable implementation as a cohesive change process (Stetler *et al.*, 2011; Harvey and Kitson, 2016). The facilitation should come from both an internal and an external perspective to provide a proper fit for the implementation process (Harvey and Kitson, 2016). Pros and cons exist when external support is excluded. For example, it can be easier to adapt any identified need for change to the context of the operation; just as some of the participants did. However, it could also be particularly difficult to carry out the implementation when the implementation leader is not prepared to make the changes or knows how to motivate them.

The unified leadership model was implemented with different solutions in the different units. Contextual factors such as organisational functioning, assistance and innovations related to intended changes need to be taken into consideration when planning for change (Durlak and DuPre, 2008). One interpretation of the various implemented solutions is that FLMs still have different prerequisites for management and leadership. The participants described that the focus in the follow-ups had been on establishing assistant FLM roles to decrease the number of subordinates per FLM, and not on improving the prerequisites for the FLMs. The evaluation of changes that have been implemented is crucial for sustainable change (Damschroder *et al.*, 2009), e.g. the identification of problems in the early stages of the process (Durlak and DuPre, 2008). The perceptions of those involved have an impact on the effectiveness of the implemented change in the organisation (Damschroder *et al.*, 2009). Therefore, such measures should be conducted. In the present study, the participants declared that they were unclear as to whether or not the changes resulted in any improvements affecting the FLMs’ prerequisites to better carry out their assignments. Furthermore, they expressed that the responsibility for follow-up evaluations rested with top management. This could suggest that the implementation leaders did not plan, coordinate or facilitate the implementation in their own operations properly, and they did not seem to monitor or evaluate the changes they themselves initiated. The participants could also have assumed the responsibility to report back to top management, as described by Urquhart *et al.* (2018). The question of whether the conditions for FLMs have improved remains unanswered.

### Methodological considerations

A grounded theory design provides explicit and sequential guidelines for conducting qualitative research, and is considered particularly well suited for exploratory investigations of social processes that have previously attracted little research attention. Caution regarding the transferability of our findings should be exercised in relation to geographical, organisational and cultural differences. Interview data were collected from one health-care organisation, where its unique system of government and the actors therein may limit the transferability to other contexts, however; they may be equally valid in other contexts. Another limitation is the modest sample recruited. It is possible that a larger sample could have added more perspectives to the theoretical model. Credibility was improved through the iterative process. These discussions and memo writing also enabled reflection on the preunderstanding that might have had an impact on the findings (Charmaz, 2006).

### Conclusion

When implementing changes, it is important that everyone in the organisation have the same understanding regarding the goal and have a shared confidence in the solutions during the entire process. In this study, we reveal that there was an initial consensus between the top management and second-line manager’s views on the need to create better conditions for FLMs, but during the process this deteriorated. At the same time, the second-line managers described the feeling of being left on their own to find contextually adapted solutions when implementing the changes. To succeed when implementing change in complex organizations, it is crucial that managers at the different levels are involved in the entire process, and that there are prerequisites established for the facilitation and achievement of the goals during the planning, implementation and follow-up.

## Figures and Tables

**Table 1.** The grounded theory result of second-line managers’ perceptions of, prerequisites for and experience from the implementation of changes in relation to the five focus areas to improve conditions for their manager’s work conditions

Relationship between categories; thematic area	Categories	Focused codes	Core category
*Engagement*	*Managers’ prerequisites – an area with an imperative need for change*	Imperative with a focus on managers’ prerequisites for work	A complex challenge with an unclear result **–** *For perceived improvement; involvement, engagement; and consideration for the context of the organisation is needed in a cohesive implementation process*
		Measures necessary for better prerequisites, irrespective of decisions	
	*Difficulties in applying content of decided directives – need to problematise*	Unproblematised decision	
		Problematising of the assistant manager’s role	
	*Engagement to create understanding – desirable to be able to take responsibility*	Uncertainties over the motive of the content from a top-down decision	
		Input was disregarded or not sought by top management	
		Lack of dialogue and participation	
*Facilitation*	*Dialog and structure as support – different needs in different organisations*	Forums for dialogue	
		Support personnel can be brought in	
		Uncertainty about support structures	
		Internal support within the organisation	
*Achievement*	*Adapting approach and strategy – to unite the needs of the organisation with the decision*	Approach to the decision	
		Adapt solution to the manager’s role in your own way to comply with the decision	
	*Are we getting where we want to go? – experiences and achievement of goals*	Experience from changes	
		Uncertainty regarding improvement of managers’ prerequisites	
